# Response to Letter to the Editor^☆^

**DOI:** 10.1016/j.ctro.2025.101002

**Published:** 2025-07-03

**Authors:** Eric D. Ehler, Grace H. Hutchinson, Jianling Yuan, Kathryn E. Dusenbery, On behalf of the authors

**Affiliations:** Department of Radiation Oncology, University of Minnesota, United States

To the Editor,

We thank the correspondent for their thoughtful and collegial engagement with our recent work, “Predicting late renal toxicity using a two-component repair model among pediatric patients receiving total body irradiation for stem cell transplant.” We are encouraged by their recognition of the model’s translational potential and appreciate the opportunity to comment on the proposed refinements.

1 Repair Kinetics and Parameterization

We acknowledge that the use of murine-derived repair parameters carries limitations. The repair half-times applied in our model were selected based on available sublethal damage kinetics data and serve as a starting point for modeling temporal repair in the absence of pediatric-specific data. We welcome future efforts to refine these parameters using clinically validated, human pediatric data sources. In our previous study modeling pulmonary toxicity in pediatric HCT [1], we similarly highlighted the need for biologically informed modeling that accounts for tissue-specific and age-specific differences.

2 Toxicity Endpoint Stratification

We explicitly acknowledged this endpoint variability and its potential impact on model precision. In our current study, we included toxicities deemed clinically significant, as defined by each source paper, to retain sufficient statistical power across heterogeneous datasets. As highlighted in our previous work on pulmonary toxicity, we strongly support the adoption of CTCAE v5.0 with Grade ≥3 stratification as a standard approach for prospective clinical outcomes reporting.

3 Low Dose Rate Effects

We agree that dose rate is a critical consideration in TBI modeling. While our two-component model accounts for temporal repair kinetics, it does not explicitly model endothelial-specific radiosensitivity or microvascular injury thresholds. These pathophysiological processes may contribute to radiation nephropathy. We support the suggestion to incorporate these processes as data become available.

We thank the correspondent again for their constructive feedback and for highlighting key considerations in the evolution of predictive models in pediatric radiotherapy. Their suggestions align well with many of the directions we are pursuing in ongoing work.

## Declaration of Competing Interest

The authors declare that they have no known competing financial interests or personal relationships that could have appeared to influence the work reported in this paper.
